# Influence of severity of illness on neutrophil gelatinase-associated lipocalin performance as a marker of acute kidney injury: a prospective cohort study of patients with sepsis

**DOI:** 10.1186/s12882-015-0003-y

**Published:** 2015-02-13

**Authors:** Jill Vanmassenhove, Griet Glorieux, Norbert Lameire, Eric Hoste, Annemieke Dhondt, Raymond Vanholder, Wim Van Biesen

**Affiliations:** Renal Division, Ghent University Hospital, Ghent, Belgium; Intensive Care Unit, Ghent University Hospital, Ghent, Belgium; Research Foundation Flanders, Flanders, Belgium

**Keywords:** Sepsis, Acute kidney injury, NGAL

## Abstract

**Background:**

The role of neutrophil gelatinase-associated lipocalin (NGAL) as a diagnostic marker for acute kidney injury (AKI) in sepsis is still debated. We hypothesized that in sepsis, the performance of serum(s) and urinary(u) NGAL can be negatively impacted by severity of illness and inflammation, and that both uNGAL and sNGAL levels can be increased regardless of presence of AKI.

**Methods:**

One hundred and seven patients with sepsis were included. uNGAL and sNGAL were measured at admission (T0) and 4 hours (T4) and 24 hours later (T24). Transient and intrinsic AKI were respectively defined as AKI according to RIFLE during the first 72 hours that did or did not recover to “no AKI” in the following 72 hours. Patients were classified according to tertiles of CRP and APACHE II score increase. The relationship between sNGAL and uNGAL was assessed by linear regression.

**Results:**

Fifty-seven patients developed transient and 22 intrinsic AKI. Prevalence of transient and intrinsic AKI were higher in patients with versus without septic shock (OR (95% CI):3.3(1.4-8.2)). uNGAL was associated with sNGAL, and this with parallel slopes but different intercepts for AKI (Y = 0.87*X + 314.3,R^2^ = 0.31) and no AKI (Y = 0.87*X + 20.1,R^2^ = 0.38). At T4, median uNGAL and sNGAL levels were higher in septic patients with versus without shock but this is independent of AKI ((545 ng/mL vs 196 ng/ml for uNGAL and 474 ng/ml vs 287 ng/ml for sNGAL (both P = 0.003)). Both uNGAL and sNGAL levels increased with tertiles of CRP and APACHE II score increase.

**Conclusions:**

Serum and uNGAL levels are influenced by severity of illness and inflammation, and this was found to be independent of the presence of AKI. There is a strong correlation between sNGAL and uNGAL levels in patients with sepsis, indicating that increased levels of uNGAL can also be due to overspill from the systemic circulation, blurring the discriminative value of NGAL as a biomarker for AKI in patients with sepsis.

## Background

Septic acute kidney injury (AKI) is associated with worse outcome compared to non-septic AKI and is regarded as a distinct clinical entity [1]. The unacceptably high mortality rates associated with septic AKI are partly explained by an incomplete understanding of the pathophysiology and a delay in diagnosis [2-6]. Early diagnosis of septic or non-septic AKI remains cumbersome because it relies on imperfect parameters such as serum creatinine while introduction of new serum and urinary biomarkers could hypothetically allow earlier diagnosis and better prognostication [7-9]. At present, NGAL (neutrophil gelatinase-associated lipocalin) has been the most frequently investigated biomarker for early diagnosis of AKI [10]. In humans, three different forms of NGAL can be found, namely a 25 kDa monomer, a 45 kDa dimer and a 135 kDa heterodimer, covalently conjugated with gelatinase [11-13]. Up till now, no commercially available immunoassays are able to make a clear discrimination between the monomer, mainly released from tubular epithelial cells, and the dimer, originating from neutrophils [12].

Some studies found increased urinary NGAL levels in patients classified as having transient AKI, suggesting presence of subtle tubular structural injury [14,15]. In addition, studies where there was a rise in biomarker level (either in serum or urine) without a rise in serum creatinine or a decrease in urinary output, resulted in speculation on the existence of a new entity called *subclinical AKI* [16,17]. However, serum NGAL levels can be increased in many other conditions beside acute kidney injury, such as inflammation [18]. As serum NGAL is filtered at the glomerular level, also urinary NGAL can potentially be influenced by inflammation [19-21].

The present study aims to characterize the origin of the raised serum and urine levels of NGAL in septic patients. We hypothesized that, as in sepsis patients, the prevalence of AKI is related to severity of sepsis, which in turn is associated with an increase in urinary and serum NGAL levels, a correlation between both urinary and serum NGAL, and severity of illness could exist, independent of the presence of AKI.

## Methods

### Study cohort

One hundred and seven consecutive patients with sepsis, admitted to the Ghent University Hospital between 12/01/2010 and 05/09/2010, were prospectively enrolled. Sepsis, severe sepsis or septic shock were defined according to the American College of Chest Physicians/Society of Critical Care Medicine Consensus Conference guidelines [22]. Briefly, sepsis was defined when two or more of the following conditions were present as a result of infection: 1) temperature >38° or < 36°, 2) heart rate >90 beats/min, 3) respiratory rate >20 breaths/min or PaCO2 < 32 mmHg (<4,3 kPa) or 4) white blood cell count >12000 cells/mm^3^ or <4000 cells/mm^3^, or >10% immature (band) forms. Severe sepsis was defined as sepsis associated with organ dysfunction, hypoperfusion or hypotension. Sepsis with shock was defined as sepsis with hypotension despite adequate fluid resuscitation or vasopressor need. Since only four patients were not classified as having either severe sepsis or septic shock, we combined sepsis and severe sepsis in a new cumulative category ‘sepsis without shock’, as opposed to ‘sepsis with shock’. Exclusion criteria were 1) a history of liver and/or kidney transplantation, 2) ICU stay less than 24 hours, 3) patients treated with chronic haemodialysis and 4) age <17 years.

Fluid management and decision-making for need of RRT were done by intensivists, who were blinded to the study, and according to protocols applied in the study hospital. The study was approved by the ethical committee of the Ghent University Hospital. Written informed consent was obtained from the patient or their next of kin. Research adhered to the tenets of the Declaration of Helsinki.

### Study definitions

We defined AKI based on the worst of either serum creatinine or urinary output criteria according to RIFLE [23]. The urinary output criterion was based on 6-hour blocks, as described by Macedo et al. [24]. Baseline serum creatinine was based on the most recent value before admission or was estimated with the MDRD equation if the latter was not available [23].

Transient acute kidney injury was defined as presence of AKI according to RIFLE, occurring in the first three days after admission and returning to no AKI within the following 72 hours. Intrinsic AKI was defined as presence of AKI according to RIFLE in the first three days of admission that did not improve to no AKI in the following 72 hours. Patients who left ICU before 5 days after admission were followed up at the department they were transferred to.

### Sample collection

Urine and blood samples were collected at the moment of admission (T0), four hours later (T4) and 24 hours later (T24). Blood samples were centrifuged at 1500 g for 10 minutes within 20 minutes after collection, and serum was aliquoted and stored at −80°C for later batch analysis. Urine was collected in a sterile manner and centrifuged at 500 g for 10 minutes, and urine samples were aliquoted and stored at −80°C for later batch analysis.

Serum and urinary neutrophil gelatinase-associated lipocalin (NGAL) were measured using an ELISA kit (Bioporto^R^ Diagnostics Denmark).

### Data collection

After informed consent, demographics and medical history were obtained. Laboratory and clinical data were registered in a dedicated database. APACHE II scores were calculated over the first 24 hours of admission.

Patients were classified according to tertiles of serum C-reactive protein (CRP) levels (CRP ≤20.10 mg/dL, CRP 20.11-30.70 mg/dL and CRP ≥30.71 mg/dL) and APACHE II score increase (APACHE II <20, APACHE II 20–25 and APACHE II >25).

### Statistical analysis

Results are reported as medians and interquartile ranges (IQR) for continuous variables, unless otherwise specified. Discrete variables are reported as numbers and/or percentages. All statistical analyses were performed using SPSS® 19. All consecutive patients fulfilling the inclusion criteria were included, irrespective of their course or duration of stay at ICU.

Demographic characteristics of the study cohort were compared using using Mann–Whitney U test (two groups) or Kruskal Wallis (>two groups) (continuous variables not normally distributed). Student’s t test (two groups) or one-way ANOVA (>two groups) were used to compare means (continuous variables with normal distribution).

As planned, patients were classified according to sepsis status (sepsis without vs sepsis with shock), AKI status (no AKI vs transient AKI vs intrinsic AKI) and tertiles of CRP and APACHE II score for comparison.

Dichotomous variables were compared between groups using Chi square analysis.

Regression analysis was used to assess association between serum and urinary NGAL.

## Results

Demographics and clinical background of the 107 included patients are presented in Tables 1 and 2 as partially published elsewhere [25] (Tables 1 and 2). Sepsis without shock was present in 42 (39.3%) patients and sepsis with shock in 65 (60.7%) patients. Twenty-eight (26.2%) patients were classified as having no AKI versus 57 (53.3%) and 22 (20.6%) as having transient and intrinsic AKI, respectively. Median APACHE II score was 21 in sepsis patients without shock and 23 in those with shock (P = 0.22) and increased from no AKI over transient AKI to intrinsic AKI (P = 0.08).

**Table 1:** **Clinical and demographic characteristics of the cohort comparing no-AKI vs transient AKI vs intrinsic AKI**

	**no AKI (n = 28)**	**Transient AKI (n = 57)**	**Intrinsic AKI (n = 22)**	**p value**
Gender male (%)	51.7	54.4	59.1	0.93
Age (years,mean/sd)	55.4(17.3)	62.6(13.2)	63.1(14.9)	0.08
Reason for admission (%):				0.11
Respiratory	46.4	38.6	36.4	
Abdominal	21.4	33.3	36.4	
Urinary	7.1	8.8	0	
Endocarditis	3.6	7.0	0	
Neurological	7.1	0	4.5	
Catheter	10.7	1.8	0	
Other	3.6	10.5	22.7	
CKD on admission (eGFR according to MDRD <60 mL/min/1,73 m2) (%)	10.7	8.8	9.1	1
APACHE II score on the first day of admission	21(10)	22(8)	24.5(9)	0.08
Fluid balance first 24 hours (liter, mean/sd)	2.2(1.8)	3.1(1.9)	5.4(2.7)	<0.001
Use of diuretics on the first day of admission (%)	7.1	14	22.7	0.29
RRT need during ICU stay (%)	0	1.8	59.1	<0.001
Vasopressor use (%)	32.1	61.4	86.4	<0.001
Total dose of noradrenaline first 24 h in μg/kg/min (mean/sd)	0.05(0.11)	0.09(0.12)	0.20(0.22)	0.001
Maximum dose of noradrenaline during first 24 h in μg/kg/min (mean/sd)	0.12(0.23)	0.25(0.31)	0.54(0.55)	0.008
Need for ventilation during ICU stay (%)	39.3	50.9	86.4	0.002
LOS in the ICU (days)	5(6)	5(10)	38(32)	0.014
ICU mortality (%)	21.4	15.8	54.2	0.002
Mortality at 90 days (%)	28.6	24.6	59.1	0.012

**Table 2:** **Clinical and demographic characteristics of the cohort comparing sepsis patients without vs with shock**

	**Sepsis without shock (n = 42)**	**Sepsis with shock (n = 65)**	**p value**
Gender male (%)	50	60	0.31
Age (years,mean/sd)	57.6(15.6)	62.9(14.3)	0.16
Reason for admission (%):			0.02
Respiratory	47.6	35.4	
Abdominal	14.3	41.5	
Urinary	14.3	1.5	
Endocarditis	2.4	6.2	
Neurological	4.8	1.5	
Catheter	4.8	3.1	
Other	11.9	10.8	
CKD on admission (MDRD <60 mL/min/1,73 m2) (%)	16.7	4.6	0.04
APACHE II score on the first day of admission	21(9)	23(9)	0.22
AKI (transient or intrinsic) (%)	25(59.5)	54(83.1)	0.007
Fluid balance first 24 hours (liter, mean/sd)	2.06(2.21)	3.8(2.58)	<0.001
Use of diuretics on the first day of admission (%)	9.5	16.9	0.28
RRT need during ICU stay (%)	0	21.5	0.001
Total dose of noradrenaline first 24 h in μg/kg/min (mean/sd)	N/A	0.17(0.16)	N/A
Maximum dose of noradrenaline during first 24 h in μg/kg/min (mean/sd)	N/A	0.44(0.41)	N/A
Need for ventilation during ICU stay (%)	39.3	73.8	<0.001
LOS in the ICU (days)	4(6)	7(16)	0.03
ICU mortality (%)	14.3	32.3	0.04
Mortality at 90 days (%)	19.0	41.5	0.02

More sepsis patients with shock versus without shock had transient or intrinsic AKI (35/65 vs 22/42 and 19/65 vs 3/42, respectively, P = 0.007). There was also an increasing positive fluid balance, need for ventilation, length of ICU stay and mortality from no AKI over transient AKI to intrinsic AKI and in patients with versus those without shock. Fourteen patients needed RRT (Tables 1 and 2).

**Figure 1:**
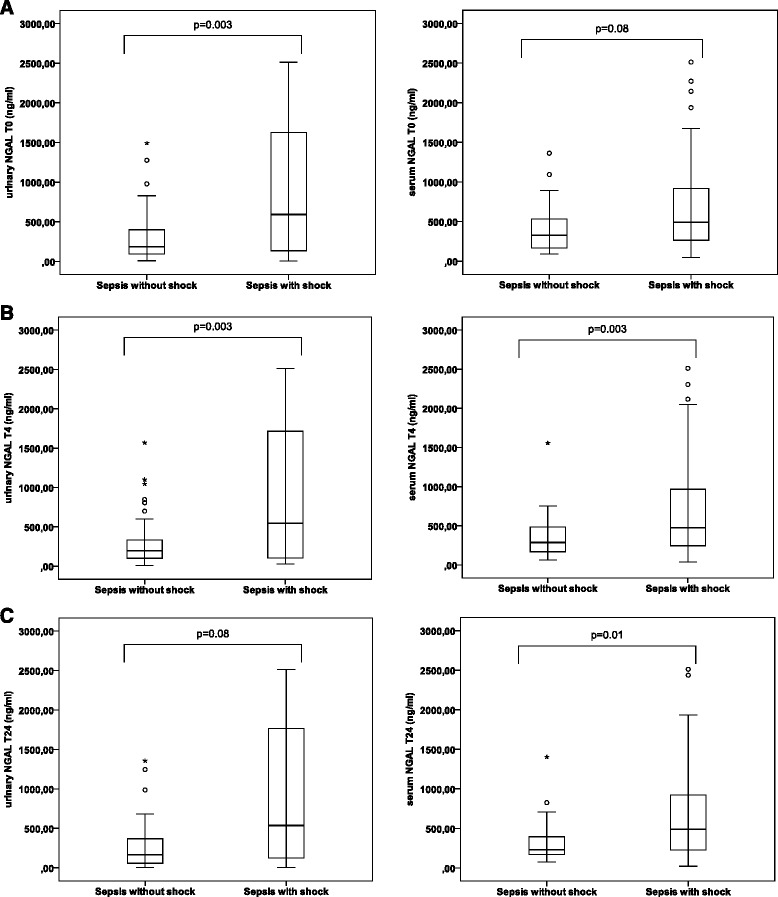
**Urinary and serum NGAL in sepsis without vs with shock. A**: Urinary NGAL (ng/mL) at time point T0 is higher in sepsis with vs without shock (P = 0.003). There is a trend for higher serum NGAL levels (ng/mL) in sepsis with vs without shock (P = 0.083). **B**: Serum and urinary NGAL (ng/mL) at time point T4 are higher in sepsis with shock vs without shock (both P = 0.003). **C**: Serum NGAL (ng/mL) at time point T24 is higher in sepsis with vs without shock (P = 0.011). There is a trend for higher urinary NGAL levels (ng/ml) in sepsis with vs without shock (P = 0.082). (° = outliers; * = extreme outliers (> three times the height of the boxes)).

**Figure 2:**
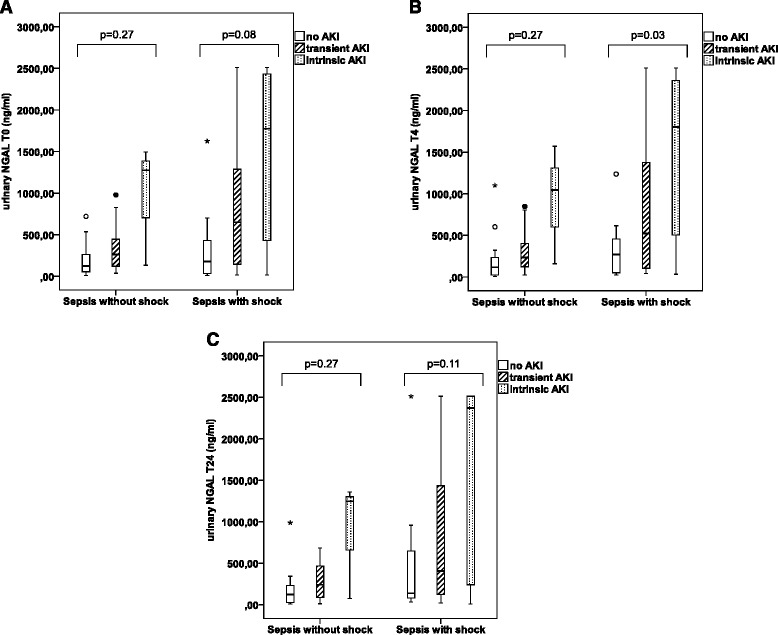
**Urinary NGAL in no AKI, transient AKI and intrinsic AKI, stratified according to sepsis severity. (A)** At time point T0, urinary NGAL(ng/ml) is not significantly different between no-AKI, transient AKI and intrinsic AKI in sepsis without shock (P = 0.27) and sepsis with shock (P = 0.08). **(B)** At time point T4, urinary NGAL(ng/ml) is not significantly different between no AKI, transient AKI and intrinsic AKI in sepsis without shock (P = 0.27). **(C)** At time point T24, urinary NGAL (ng/mL) is not significantly different between no-AKI, transient AKI and intrinsic AKI in sepsis without shock (P = 0.27) and sepsis with shock (P = 0.11). (° = outliers; * = extreme outliers (> three times the height of the boxes)).

Median urinary and serum NGAL levels were higher in sepsis patients with versus those without shock (Figure 1A, B, C and Table 3). All no AKI patients had serum NGAL levels above the generally accepted cut-off of 150 ng/mL [26,27] at all time points. All sepsis patients with shock had urinary NGAL levels above 150 ng/mL at admission and four hours later, even if they did not have AKI. Urinary and serum NGAL levels were higher in intrinsic versus transient and no AKI patients, but there was substantial overlap limiting discriminative value (Table 3). When classified according to sepsis without versus with shock, discriminative value of NGAL for AKI further decreased (Figure 2A, B and C).

**Table 3:** **Urinary NGAL and serum NGAL (ng/mL) in no AKI vs transient and intrinsic AKI**

	**Sepsis without shock**				**Sepsis with shock**				
	**No AKI (n = 17)**	**Transient AKI (n = 22)**	**Intrinsic AKI (n = 3)**	**p value**	**No AKI (n = 11)**	**Transient AKI (n = 35)**	**Intrinsic AKI (n = 19)**	**p value**	**Overall p value**
uNGAL T0	125(262)	262(350)	1276	0.27*	178(457)	649(1164)	1775(2108)	0.08*	0.001^#^
uNGAL T4	116(256)	234(310)	1044	0.27*	269(511)	523(1384)	1802(2046)	0.03*	0.009^#^
uNGAL T24	122(218)	239(383)	1245	0.27*	137(612)	405(1523)	2372(2308)	0.11*	0.084^#^
sNGAL T0	220(269)	288(477)	235	0.44*	290(221)	493(579)	962(894)	0.003*	0.03^#^
sNGAL T4	218(193)	336(338)	284	0.17*	267(192)	469(604)	975(940)	0.011*	0.002^#^
sNGAL T24	203(189)	295(364)	184	0.44*	283(188)	475(435)	1052(670)	0.001*	0.001^#^

*P-values refer to the difference between no AKI, transient AKI and intrinsic AKI, separately for patients with vs without shock.

^#^P-values refer to the difference between no AKI, transient AKI and intrinsic AKI either with or without shock at each time point.

**Figure 3:**
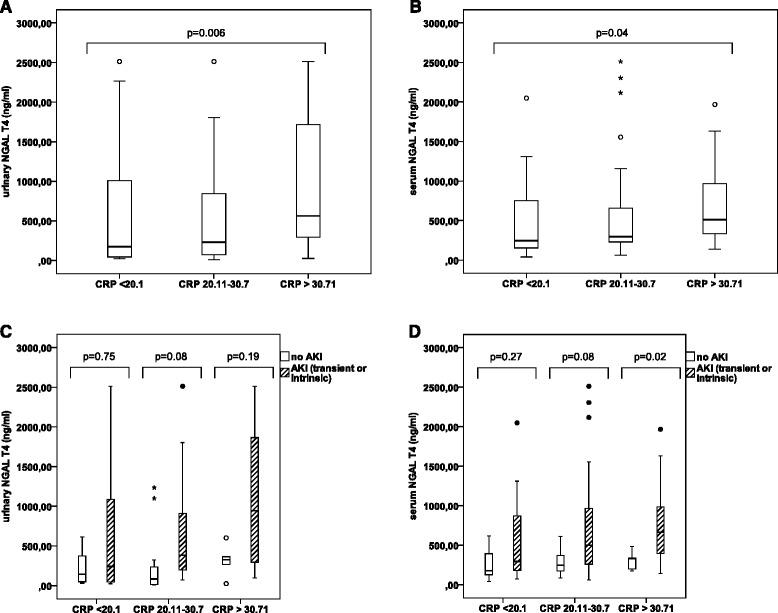
**Influence of inflammation on serum and urinary NGAL. (A)**: Urinary NGAL (ng/mL) increases together with increasing levels of CRP. **(B)**: Serum NGAL (ng/mL) increases together with increasing levels of CRP. **(C)**: Urinary NGAL (ng/mL) is not significantly different between AKI (transient or intrinsic) and no AKI, when stratified according to tertiles of CRP increase. **(D)**: Serum NGAL (ng/mL) is not significantly different between AKI (transient or intrinsic) and no AKI in the two lower tertiles of CRP increase. (° = outliers; * = extreme outliers (> three times the height of the boxes)).

Urinary and serum NGAL levels increased with tertiles of CRP (175 ng/mL vs 229 ng/mL vs 563 ng/mL, for uNGAL and 245 ng/mL vs 296 ng/mL vs 512 ng/mL for sNGAL (P = 0.006 and P = 0.04, respectively) (Figure 3A and B). Neither uNGAL or sNGAL had a discriminative value for differentiating AKI (transient or intrinsic) from no AKI (Figure 3C and D).

**Figure 4:**
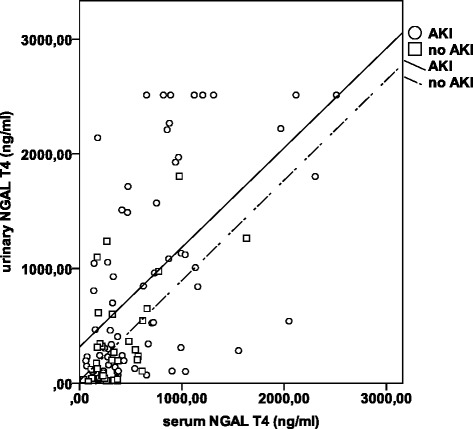
**Linear regression between serum NGAL and urinary NGAL in no AKI and AKI.** For no AKI: y = 0.87*x + 20.1 and for AKI: y = 0.87*x + 314.3. R^2^ for AKI = 0.31 and R2 for no-AKI = 0.38 (P <0.001).

We found a strong correlation between sNGAL and uNGAL, both in patients without and with AKI (R^2^ = 0.38 for no AKI and R^2^ = 0.31 for AKI), but with different relationships in no AKI (Y = 0.87*X + 20.1) versus AKI (Y = 0.87*X + 314.3), respectively (P < 0.001). The slopes of the regression lines followed a parallel course (Figure 4).

Analyses of the correlation between sNGAL and uNGAL levels at the other time points demonstrated comparable findings (data not shown).

**Figure 5:**
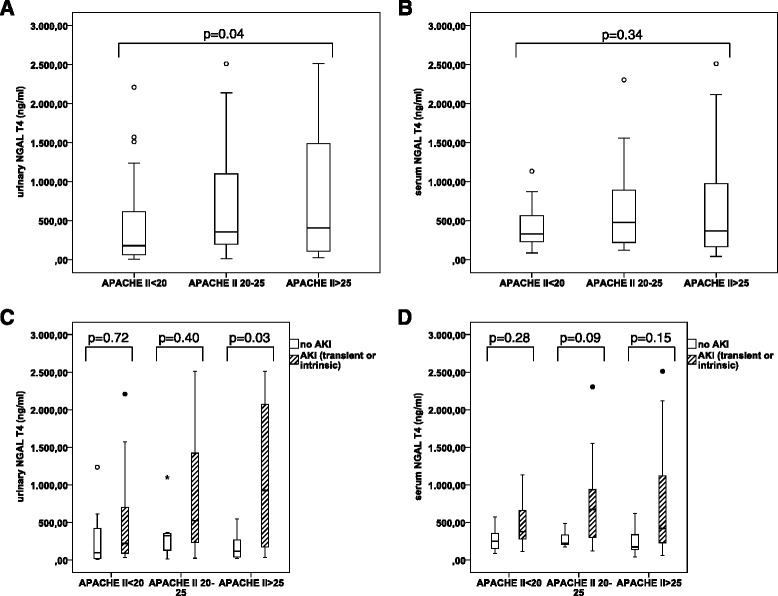
**Influence of severity of illness on serum and urinary NGAL. (A)**: Urinary NGAL (ng/mL) increases together with tertiles of APACHE II score increase. **(B)**: There is a trend for increasing serum NGAL (ng/mL) together with APACHE II score increase, in the two lower tertiles. **(C)**: Urinary NGAL (ng/mL) is not significantly different between AKI (transient or intrinsic) and no AKI, in the two lower tertiles of APACHE II score increase. **(D)** Serum NGAL(ng/ml) is not significantly different between AKI (transient or intrinsic) and no AKI, when stratified according to tertiles of APACHE II score increase. (° = outliers; * = extreme outliers (> three times the height of the boxes)).

There was also a correlation between the APACHE II score and uNGAL (P = 0.002, P <0.001 and P = 0.003 at T0, T4 and T24) and between the APACHE II score and sNGAL (P = 0.007, P = 0.003 and P = 0.07 at T0,T4 and T24). Median urinary NGAL levels increased with increasing tertiles of APACHE II (179 ng/mL vs 355 ng/mL vs 405 ng/mL for APACHE II <20, 20–25 and >25 respectively, P = 0.04) (Figure 5). There was an increasing trend in serum NGAL levels over the first two tertiles (Figure 5).

## Discussion

In this cohort of septic ICU patients, we confirmed that the risk for AKI increased with severity of sepsis. Serum and urinary levels of NGAL increased with severity of illness and inflammation, as assessed by APACHE II and CRP. In addition, there was a strong correlation between urinary and serum levels of NGAL, irrespective of presence of AKI. Although there was a significant difference in uNGAL levels between no AKI, transient AKI and intrinsic AKI, this difference did not remain when patients were stratified according to severity of sepsis (sepsis with or without shock) and to tertiles of CRP or APACHE II score increase, except for sNGAL in the highest CRP tertile where a significant difference between AKI and no AKI could be found. However, across all tertiles, there was an important overlap in NGAL levels between AKI and no AKI, the result being that NGAL is not a reliable biomarker to discriminate between AKI and no AKI in the individual case.

It remains unclear whether in sepsis patients, increased urinary NGAL is a marker of structural tubular injury, the result of overspill from the systemic circulation or just a marker of severity of illness. This is also emphasized by the fact that no commercially available assays make a distinction between ‘renal’ NGAL and ‘neutrophil’ NGAL. Cai et al. confirmed that NGAL is present in different forms in the urine by using five ELISAs (using different monoclonal and polyclonal antibodies) and one polyclonal-based RIA [11]. Authors concluded that it should be possible to construct an assay that preferentially identifies NGAL originating from the tubular epithelium or the neutrophils. Martensson et al. demonstrated that the ratio of two ELISAs, each using a different monoclonal antibody, could detect the monomeric form (which mainly originates from renal tubular cells) with an AUC ROC of 0.92 in ICU patients without CKD [13]. However, more recently, Glassford et al. assessed monomeric and dimeric uNGAL contribution using western blotting-validated enzyme-linked immunosorbent assays and their calculated ratio in 102 patients with SIRS and found that at best, urinary forms of NGAL are fair predictors of renal or patients outcome. The authors conclude that the nature and source of uNGAL are complex and challenge the utility of NGAL as a uniform biomarker [12].

NGAL is filtered into the primary urine and almost completely reabsorbed by the tubular epithelium via the megalin receptor under normal circumstances [28]. One of the concerns of using NGAL as a biomarker for AKI is that even in the absence of AKI, NGAL levels can increase during inflammation [18,29-33]. We demonstrated that in patients with sepsis, serum NGAL levels increase in parallel with the severity of sepsis, severity of illness and severity of inflammation and thus increased levels might not automatically reflect tubular damage.

High levels of serum NGAL can overwhelm the reabsorbing capacity of the proximal tubule so that urinary NGAL levels might increase, even in the absence of structural tubular injury. We found a strong correlation between serum and urinary NGAL levels, both in patients without and with AKI. The value of urinary NGAL for differentiating between AKI and no AKI was also low due to overlap between the two groups, irrespective of severity of illness, sepsis or inflammation.

These findings underline that the concept of “subclinical AKI” should be used with caution, however, they do not contradict the existence of this concept in certain patients. Indeed, urinary NGAL was well correlated with serum NGAL (R^2^ = 0.37), but the regression line went through the origin for no AKI patients, whereas it did not in AKI patients. This suggests that in AKI, there is some degree of either local tubular production or reduced reabsorption, both of which may reflect tubular injury.

In any case, our data indicate that the suggestion to use uNGAL as a diagnostic marker because of its allegedly discriminating role in differentiating AKI from no AKI should be carefully considered, at least in patients with sepsis. In addition, when including patients with ‘subclinical AKI’, based on NGAL positivity only, in an interventional trial, those interventions that only focus on preventing or healing tubular injury risk to be less useful. The latter because, in a substantial part of patients, the increased urinary NGAL will not be the result of tubular damage but rather of overspill from the circulation as a consequence of high circulating levels induced by inflammation.

Designing an immunoassay that perfectly discriminates between monomer and dimer NGAL will only partly solve this issue. Since the common pathway in CKD relates to tubulointerstitial damage which in turn could imply high levels of monomer NGAL, a tool that allows the measurement of monomer NGAL only, would not be helpful in differentiating between AKI, acute on chronic kidney disease and CKD. Also, the role of leukocytes in the pathophysiology of septic AKI is being increasingly recognized [34]. So the presence of dimer NGAL can also be an indication of local tubular damage rather than just being a sign of glomerular overflow or presence of leukocyturia in the context of urinary infection.

A limitation of this observational study is that it describes a relatively small cohort of septic patients. However, to our knowledge, this is the first study providing information on prospectively collected serum and urinary NGAL levels at different time points during the first 24 hours after admission in septic patients.

## Conclusions

In patients with sepsis, levels of urinary and serum NGAL and the prevalence and severity of AKI are strongly associated with severity of illness and inflammation as expressed by APACHE II and CRP. There is a strong correlation in sepsis patients between serum and urinary levels of NGAL, irrespective of presence of AKI. Therefore, the conclusion that presence of NGAL in the urine implies tubular injury should be made very cautiously in sepsis patients.

## Abbreviations

AKI: Acute kidney injury
APACHE II score: Acute physiology and chronic health evaluation II score
CRP: C-reactive protein
CKD: Chronic kidney disease
eGFR: Estimated glomerular filtration ratio
ICU: Intensive care unit
LOS: Length of stay
MDRD: Modification of Diet in Renal Disease
NGAL: Neutrophil gelatinase-associated lipocalin
RIFLE: Risk, Injury, Failure, Loss of kidney function and End-stage renal disease
RRT: Renal replacement therapy
